# Hospital and Institutional News

**Published:** 1913-04-12

**Authors:** 


### HOSPITAL AND INSTITUTIONAL NEWS.

### MR. MASTERMAN'S PROMISE OF AMENDMENT.

Speaking at the inaugural dinner of the Faculty of Insurance on Saturday last, Mr. Masterman, Financial Secretary to the Treasury, said that if it could be shown how the Insurance Act could be improved, no pride in their offspring would prevent their acceptance of amendments. "The Chancellor of the Exchequer and I," he is reported to have added, "will shortly introduce an amending Bill, and any suggestion for improving the administration will be welcomed." He was confident that the National Insurance system was going to be a permanent part of the life of this country. It might be that life insurance would in the future be added to sickness insurance; that the tentative campaign, introduced through the Insurance Committees, against conditions which made for the prevalence of disease, such as bad housing and overcrowding, might be developed beyond the dreams of any one present that night; and that the comparatively simple method by which through the Act they were subsidising medical research might be developed beyond the expectations even of those who had placed it in the Act. What precisely these visionary speculations may mean we do not know, nor apparently, if we may judge from his elaborately vague language, does Mr. Masterman himself. An amending Bill to an ill-considered and hasty Act can hardly, we should hope, seek to get its unfortunate predecessor into further trouble by again rushing through far-reaching "reforms."

### PANEL SERVITUDE REPUDIATED IN IRELAND.

The question of fees for certification is still actively engaging the medical profession in Ireland, and the opposition to the terms of the Insurance Commissioners is universal. Without exception, we learn, the practitioners have refused to join the county panels until a fee of three shillings per head for certification is assured to them. Meetings have been held throughout Clare, Longford, and Wicklow, at which the medical men declined to form panels, as a protest against the fee offered by the Insurance Commissioners which they regard as totally inadequate. The certain victory which awaits the profession provided that it maintains a united front, coupled with the example of solidarity set by the Cork hospitals on another Insurance question, also referred to in these Notes this week, should not be lost on the profession in England, who with three months of panel servitude behind it has had sufficient experience of its evils to make a determined attempt to acquiesce in them no longer.

### INCREASED PAY FOR MENTAL WORKERS.

Mental hospital workers will be glad to learn that a report recommending a revised scale of salaries and emoluments for certain classes was presented by the Finance Committee to the Metropolitan Asylums Board last week. If the proposed alterations be carried out, the increase in salaries is understood to represent an addition of £20,000 a year. Resolutions to adopt the proposals will be moved at a future meeting of the Board. The suggestions for an increase are urged on the ground that the cost of living has increased of late years, and the Finance Committee has been instructed to report on the matter in relation to certain staple articles of diet. The matter is important from the fact that many mental workers live out of the institutions, and their living-out allowance must naturally be affected by any change in the cost of living. It is also contended that the increased cost of living is recognised in the calculations which guide the Board in awarding pensions. The Municipal Employees' Association has been interesting itself in the matter. The strain of mental work on all classes of workers is so great that they may reasonably hope for better conditions of work and increased pay; and institutional workers generally, who lately had the opportunity of studying Dr. F. A. Elkins' remarks on the "Living-out System at Leavesden" in our columns, will be in a position to sympathise with their colleagues.

### THE NATIONAL MEDICAL GUILD.

The distracted counsels which proved the undoing of the British Medical Association in its dealings with the Chancellor last winter will evidently not be encouraged in the newly formed National Medical Guild, which is now appealing for support from the medical profession. The first President of the Council is Dr. Buttar, well known for his activities in the West of London in connection with the British Medical Association, and the General Secretary is Mr. P. C. Raiment, prominent for the last year or two in connection with the agitation against service under the Insurance Act. The Guild is applying for registration under the Trade Union Act, a tactical measure which confers very considerable advantages in the way of protecting its funds. It is clear from Article II. of the rules that the scope of the Guild's endeavours will largely exceed that of the British Medical Association, for it is expressly stated that political propaganda and the promotion of legislation are to be engaged in. Another object of the Guild is to provide legal assistance for members, thus trenching somewhat on the province of the Defence Societies; this, it may be recalled, the British Medical Association has always declined to undertake, though proposals to that effect have been considered more than once in the past. We have quoted enough to show that the National Medical Guild aims at protecting and helping the profession of medicine and its practitioners in a way that no single existing organisation has done or can do. As a new society struggling for elbow-room, it has disadvantages to contend against; but we do not doubt that the translation of its trenchant policy into deeds equally vigorous will soon bring it such an accession of membership as will render it a formidable agency in promoting its avowed aims and objects. The present offices of the Guild are at 34 Villiers Street, Strand, W.C.

### THE SATURDAY FUND ON THE ALMONER SYSTEM.

The delegates of the Hospital Saturday Fund met last Saturday to consider the Report of the Special Committee *re* Inquiries at Hospitals and the Treatment of Insured Persons, which they had appointed on January 4, 1913, to report upon the Report of the Distribution Committee as to the reception and treatment of subscribers and their dependants at the voluntary hospitals. The idea was to consider the above in view of the Report of the Committee appointed by King Edward's Hospital Fund on Out-patients, and, secondly, having regard to the changed circumstances arising from the National Insurance Act, 1911. The Special Committee have met four times, and submitted the following, here abbreviated, report: "Hospital Almoners' System.—While recognising that the hospitals have introduced the almoner system for the purpose of preventing abuse and assisting patients, and while believing that the system may be beneficial in some respects if carried out with care and sympathy, the Hospital Saturday Fund considers that such a system is unnecessary for, and inapplicable to, its supporters and their dependants. The Fund's relation to the work of the hospitals, and the grants which it makes in support of that work, entitle it to special and sympathetic treatment at the hands of the hospital authorities, and it is considered that patients sent to a participating hospital with a recommendation from the Fund should be exempted from the almoners' inquiries, and should be medically treated as the case requires without such investigation. If in any case the hospital authorities consider that the recommendation of the Fund is being abused the case should be brought to the notice of the Secretary of the Fund, who will cause inquiries to be made, and report the result to the Distribution Committee." Resolutions embodying these points were recommended, including one to make it in order for the Board to refuse grants to hospitals declining to accept the Fund's recommendations without inquiry. After a discussion of nearly two hours the debate was adjourned for six weeks.

### A DOCTOR AWARDED DAMAGES FOR SLANDER.

Dr. Agnew, of Blundellsands, was the plaintiff in three actions for slander, which came before Mr. Justice Bankes at the Liverpool Assizes last week. The defendants were six residents in Blundellsands, two of whom were ordered to pay £300 damages to a lady in the neighbourhood in another action for slander in which Dr. Agnew's name was coupled improperly with that of the plaintiff. In Dr. Agnew's three actions it was stated that he had been ruined mentally, physically, and professionally through the effects of gossip in his neighbourhood. All three actions were settled on the defendants agreeing to pay between them sums amounting to £1,250. They also made an apology to Dr. Agnew and admitted that there was no justification for the words which they had used. Mr. Justice Bankes said that he hoped that the result of the actions would lead people to realise the pain and suffering, apart from financial consequences, which is caused by irresponsible gossip. We cordially concur in this hope, and trust that, though £1,250 cannot repay the mental anxiety and distress caused by unfounded slander, and that an apology can never wholly undo the evil effects of contumely and slanderous words, yet that the public vindication which Dr. Agnew has received may restore his mental and physical health.

### PRINCESS LOUISE AT STEPNEY DISPENSARY.

The new house of the Stepney and Mile End Dispensary for the prevention of consumption was opened by Princess Louise, Duchess of Argyll, last Saturday. This institution is one of the three which have been opened in Stepney, and is a memorial to the Princess' brother. In declaring the dispensary open Princess Louise said that she felt sure that, as dispensaries were linked up with one another in London, increasingly successful steps would be taken to banish the disease. Sir Alexander Henderson, treasurer of the Central Dispensary Fund, Sir Malcolm Morris, and Dr. Ernest Schuster were among those present. Though by no means, in spite of certain rash enthusiasts, the only or the best means for preventing the disease, there is no doubt that the co-ordinated network of dispensaries, now more or less in process of formation, will greatly aid in controlling tuberculosis in London; and that in any future scheme of co-operation between the sanatorium, the chest hospitals, and the Poor-Law infirmary's phthisical wards the dispensary must play an important part.

### A LONDON GUARDIAN ON INFIRMARY PROGRESS.

Canon Curtis, the retiring chairman of the Wandsworth Board of Guardians, reviewed at the last meeting the work accomplished during the previous three years at the infirmaries. He pointed out that St. James' Infirmary was opened by the President of the Local Government Board a few months after the present Board came into existence and to-day had 464 patients. He added that before the building of this infirmary St. John's was scandalously overcrowded, and 375 patients were boarded out in all directions. While they were contributing largely to the Metropolitan Common Poor Fund, they were for years losing their proper claim upon that Fund. St. John's was used for chronic cases and children to the number of 607. Now almost every patient was housed in the Union, and all this, he was happy to say, with no increase of the rates. They were about to lose the service of Dr. Neal. Only those who knew St. John's Infirmary a few years ago, when there was very little efficient management in the commissariat and nursing departments, when beds by the dozens had to be made up on the floors of the wards, could appreciate to the full what the Board owed to Dr. Neal, who, without a murmur or a complaint, put himself to the task imposed upon him, and with tact and management saved the Board from even a breath of scandal, which, under the circumstances, might easily have arisen had they had a less self-reliant man at the head of affairs.

### GOVERNMENT MEDDLING IN MEDICAL MATTERS.

An amusing instance of what happens if Government officials poke their noses too far into matters medical may be culled from the *Muenchener medizinische Wochenschrift* and the *Journal of the American Medical Association*. It appears that the tour in America of Dr. Friedmann, of tuberculosis fame, has interested the United States Government so much that the State Secretary forwarded to the President a report upon Dr. Friedmann's mode of treatment. This report, which comprised a verbal translation of a paper read before the Berlin Medical Society, with an introduction from the duly active and energetic American Consul-General there, was passed on to the American Senate, and actually published as a sort of Blue Book—document Number 1018 to be exact. But the American medical paper we have mentioned above is unpatriotic enough to question the value of it. "Dämpfung" or percussion dullness, appears as "oppression"; while "small bubbling râles" are rendered "rattling in the small bladder." Technical reports to non-technical bodies are always an empty kind of affair, but this one may have been a little alarming to the hypochondriacally disposed. Our famous German contemporary is quite right to notice this excess of lay zeal, but it is doubtful whether Dr. Friedmann is to blame because the police have to "move on" the crowd outside his lodging-place. However, if an active specific remedy for consumption is discovered, we do not think it will be hawked around in the style of the mediæval travelling physician.

### THE LATEST ALMONER'S APPOINTMENT.

We understand that an almoner, trained by the Hospital Almoners' Council, has been appointed to the out-patient department of the new South London Hospital for Women, which was opened last week. As the number of patients attending is not likely to be very large to begin with, the almoner will, for the present, work half-time only. It is anticipated, however, that a full-time almoner's work will be needed later.

### WORSE THAN CLUB PRACTICE.

At a meeting of the London Insurance Committee last week it was decided that, as a rule, the following classes of persons should be allowed to make their own arrangements for medical attendance and treatment for the present year: Persons the nature of whose occupation renders it impossible for them to secure the services of a particular doctor; persons who desire a special form of treatment; persons under treatment, including all cases of tuberculosis; and persons who are employees of doctors not on the panel. This recognition of the right to free choice of doctor, slender and limited as it is, appears in truer perspective when we add it was followed by a resolution put forward by Mr. F. J. Harris and seconded by Mr. E. B. Turner to the effect that the Medical Benefit Sub-Committee should report as to whether the present system of medical benefit in London could be improved, and, if so, should submit recommendations. Mr. Harris said that while a number of complaints reached the committee not one in a thousand of those cases ever reached them. The approved societies were constantly receiving complaints, and, he added, while there were before January last 1,500 medical men engaged in contract practice in London, there were now only 1,258 doctors on the London panel, who had to attend five times the number of patients which the club doctors had had to attend. Mr. Turner said that from all over the kingdom he learnt that the insured people, except perhaps in some isolated districts, were not receiving as good attendance now as they had received under the old club system. This latter statement might serve as an epitome, if it does not prove the epitaph, of panel servitude. To have provided something worse than club practice is a triumph of the disastrous legislation which party politics produce.

### NEW CHAPLAIN AT THE ROYAL INFIRMARY, BRISTOL.

The Rev. P. W. Bischoff, who has been working at St. Sepulchre's Church, Northampton, and as assistant chaplain at Northampton Hospital, has been appointed to the chaplaincy of the Royal Infirmary, Bristol. Mr. Bischoff has spent a large amount of his ministerial life among the suffering and poor, having been for some years in the slums of South London. He is, nevertheless, a keen sportsman, taking an active interest in hockey, and an expert motor-cyclist. He is also a good baritone singer.

### ANOTHER INSTITUTIONAL CHAPLAIN'S DEATH.

We have to record the sudden death of another institutional chaplain in the person of the Rev. Francis J. Greenwood, late of the St. Marylebone Infirmary, Rackham Street, W., which occurred as the result of an accident that he met with on the Central London Railway. It appears that the deceased, after having alighted from a train that was going to the depot, ran to one of the gates of the train and keeping hold of one of the door rails ran along the platform, with the result that he was crushed by the train and his body was afterwards found on the line. At the inquest the jury returned a verdict of accidental death. Mr. Greenwood was ordained in 1890 to the curacy of St. Paul's, Penge, and was appointed chaplain to the infirmary in 1893, which post he held until his death.

### THE PRODUCTIVE WORK OF THE DARENTH COLONISTS.

The work of the Darenth Industrial Colony, of which Dr. Rotherham is medical superintendent, came up for discussion at a meeting of the Metropolitan Asylums Board last Saturday. The Colony now comprises nearly two thousand patients, and was established to train and treat improvable imbeciles. It was stated at the meeting that since 1907 over £20,000 worth of work has been performed by the Colonists at a profit of over £12,000.

The Board referred to the Finance Committee the question of granting Dr. Rotherham an additional £100 a year, so long as he retains the appointment of medical superintendent, in consideration of the exceptional services which he has rendered in successfully organising the development of the Colony. Its aims and work are not as well known as they ought to be to institutional workers, but with the example of the Monyhull Colony for Epileptics before them London administrators should be glad to think that an analogous classification of mental patients has been carried out in the Metropolis.

### RAISING THE RATES BY SANATORIUM "BENEFIT."

If the promised "first-class hotels" had really been provided as a part of sanatorium "benefit," however disastrous they might have proved from a medical and institutional point of view, it would not be quite surprising if rising rates as well as rising taxation accompanied the change. But the hollowness of sanatorium benefit none the less has managed to increase local expenditure. In consequence of the absence of sanatoria large numbers of insured consumptive patients in Willesden have been sent to the infirmary, no means of institutional treatment having been provided for them, with the result that the Guardians are announced to have increased their expenditure to the extent of a penny rate. We hope that the local ratepayers will prove alert enough to see that they are not made to pay for the broken promises of party legislators.

### INSURED PERSONS' DEPENDANTS UNDER FOURTEEN.

The City Insurance Committee has addressed a letter to the Cork hospitals suggesting that in the case of children who are dependants of insured persons recommended for institutional treatment 21s. per week is not "a fair or reasonable charge," and that 12s. 6d. per week for this class of patient would be more than adequate. This letter has been laid before the Cork Joint Hospitals Committee, the position of which, we understand, has been complicated by the fact that the trustees of the North Infirmary had agreed to reduce the charge for surgical tubercular patients under fourteen years of age from 21s. to 12s. 6d. At a meeting of the Joint Hospitals Committee, before which the City Insurance Committee's letter was laid, it was unanimously decided to adhere to the agreement previously arrived at, that the charge should be 21s. per week for all surgical tubercular patients, and the secretary was instructed to write to the trustees of the North Infirmary asking them to rescind the resolution which they had passed reducing the charge for these patients to 12s. 6d. as being at variance with the agreement arrived at by consent. As all the Cork hospitals except the North Infirmary have decided to uphold the demand for the 21s., we may take it that the North Infirmary will prefer to stand by its sister institutions in this matter.

### LESSEE OF KING'S COLLEGE HOSPITAL.

The arrangements for granting a ninety-nine years' lease to Messrs. W. H. Smith and Son of the site and buildings of King's College Hospital, St. Clement Danes, have been virtually concluded, and the famous old hospital will shortly pass into commercial hands. The terms, on behalf of the governors, which have been announced by Messrs. Weatherall and Green, and the grant of a ninety-nine years' lease involve the obligation to spend £100,000 on new buildings. It will be recalled that the property comprises an island site with three frontages. It is understood that the scheme received the sanction of Mr. Justice Eve, in the High Court, on Monday, and that he confirmed the governors' proposal to accept the offer of Smith and Son. By the destruction of the old hospital buildings almost the last link with the old neighbourhood is removed. When the hospital was founded in 1839 the locality had an unfavourable reputation as the haunt of Jack Sheppard and less notorious evil-doers. It was, in fact, a congeries of slums, and its chief recommendation as a hospital site was the extreme poverty of the people who inhabited its narrow alleys. But now that Clare Market, also once of doubtful reputation, has yielded to Aldwych and Kingsway, it is natural that the northern end of one of the finest streets in London should follow suit with new buildings congruous in object and architecture with their surroundings. The improvements led to a dispersal of the crowded inhabitants and made inevitable a new site for the institution. The completion of the negotiations is, no doubt, due to the good offices of Mr. W. F. D. Smith, who has given already such invaluable help to this institution.

### FREE CHOICE OF DOCTOR IN SURREY.

The following three classes of persons have been allowed by the Surrey Insurance Committee to make their own arrangements for medical attendance and treatment for the current year: (1) Persons whose occupation makes it impossible for them to avail themselves of the panel system; (2) special cases entitled to consideration—(*a*) persons desirous of receiving homœopathic treatment, (*b*) persons desirous of receiving treatment from a woman doctor, (*c*) persons residing a considerable distance from a panel doctor; (3) domestic servants and other persons desirous of being treated by the doctor attending the household, and persons showing good cause desiring to continue receiving treatment from a doctor not on the panel. It is stated that under these headings 225 applications had been received, of which 220 have been granted.

### A STILL-BORN PROPHECY.

A rather curious commentary on the statement of Mr. J. A. Dawes, M.P., when presiding over the London Insurance Committee last week, that he had every reason to believe that "a very large number of additional doctors" were going to join the panels, has been provided by returns given at a meeting of the Middlesex Insurance Committee on Monday. It was there stated that the number of panel doctors was 520, but that only 445, or about three-quarters, had renewed their agreements. The

"very large number of additional doctors" seems—at any rate in a locality close by—to mean in practice an immediate drop of 25 per cent. For three months we have had to record the ghastly fruits of sweating medical men and the deaths of patients from inability to obtain their promised benefits. If such deaths occurred in the green tree, what shall be done in the dry? With even fewer doctors the prospect is one that may well appal an insured person who can draw the moral from these figures. The meeting of the Middlesex Insurance Committee affords another fact hardly less disquieting. It appears that approximately one-quarter of insured persons in Middlesex have not selected their doctor. What a commentary on this is afforded by this Committee's determination not to allow free choice of doctor save in exceptional circumstances! We confess that we are not surprised that the clerk reported that a full service of doctors is still unobtainable in the West district, and that the Committee has failed to induce the doctors there to join the panel.

### TOBACCO HEART AND ALCOHOLISM.

At the inquest on the late Mr. Rankine Dawson, L.R.C.P., M.R.C.S., who died suddenly in Pimlico, the medical evidence of Dr. Braxton Hicks, who made a post-mortem, showed that death was due to heart failure and was accelerated by chronic alcoholism, in accordance with which a verdict was returned. In the course of the evidence it was stated that Mr. Dawson used to smoke twelve or more cigars a day, and a surgeon who gave evidence of identification said that he had treated him for "tobacco heart." The deceased was a younger son of the late Sir J. W. Dawson, Principal of McGill University, where he graduated B.A. with honours in philosophy in 1878, and four years later in medicine. He had made a special study of children's diseases and unsuccessfully contested East Edinburgh in the Unionist interest in 1906.

### PREPARATORY SCHOOLS AND MEDICAL INSPECTION.

The suicide of a schoolboy, whose defective vision prevented his entry into the Royal Navy, has been made an excuse for reviving the general question of the suicide of schoolboys, which is increasingly complained of on the Continent, and for once more urging parents to have their sons medically examined before allowing them to assume that their future will be that of a Naval cadet. This seems such a simple and obvious way of avoiding disappointment and waste of time that it is hardly worth while mentioning were it not that it opens up the far more important question of the medical inspection of boys in preparatory schools. In spite of the fact that the parents of such boys are usually in a position to afford sufficient medical advice, and sometimes to indulge in superfluous coddling, a reasonable case for insisting on the school medical inspection of children of the middle and upper classes exists. Carelessness and stupidity are not confined to those whose incomes are below the income-tax limit, and many cases of so-called stooping, spinal curvature, and pigeon-chests could be remedied by the impartial timely examination of the private school medical inspector. But while this would do much to remedy neglected incipient forms of disease, it would be idle to hope for medical inspection to guard against suicide. Boys psychologically inclined to self-destruction will always exist, and the only way to cope with special instances is to have a system of education sufficiently elastic to embrace the exceptional as well as the average individual. The enormously extended curriculum and the unintelligent memorising of innumerable facts for examination purposes are among the causes which foster melancholia in boys.

### CITY GUARDIANS AND BART.'S

The City of London Guardians have been criticising the authorities of St. Bartholomew's Hospital for their treatment of two men sent by them to the local workhouse. This simple statement will be enough for those who have had experience of similar criticisms without going into further details. It was resolved to refer the matter to the house committee. The general moral of these occasions of friction between voluntary hospitals and Poor-Law infirmaries may, however, be stated. It is that there is no adequate channel of intercommunication between the two. Mistakes no doubt have been made by both sides in the past, but opportunities of friction will never be removed until the troublesome routine now necessary before a patient can be passed on from one institution to another is replaced by some administrative co-operation.

### A HOLOCAUST OF LEPERS.

Some weeks back the reading public were horrified by a brief telegram from China announcing a massacre of lepers. The details now received from China per mail are still more shocking. The massacre took place at Nanning, a city on the West River, at the head of its navigation, in the province of Kwangsi, and the particulars are supplied by the Roman Catholic Mission. It seems that the lepers of that city had been much harassed by the people and were compelled to take refuge in a little wood about half a mile from the suburbs. The Mission, pitying the case of these afflicted sufferers, bought land for the erection of a lazar house, and work commenced. The native merchants, who expressed themselves strongly in favour of the scheme, advised the Father-in-Charge to secure official approval, and he accordingly sought an interview at the Yamen. Posters, however, soon appeared on the walls of the town setting forth that the lepers were the "rejected of Heaven," that it was "impious to succour them," and useless to "spend money on feeding them." In the city the desirability of getting permanently rid of them was openly discussed as a measure of public utility. The worthy priests of the Mission, alarmed at rumours of intended cold-blooded violence towards the lepers, called upon the provincial President and pleaded their cause. They were politely received and their proposal to erect a lazar house was commended; but the President added that the site should be further from the city, and that he would give orders to the Prefect to provide one. On the morning of December 14 thirty-nine of the lepers were inhumanly put to death by order of the same man who had blandly praised the charitable action of the missionaries. A body of soldiers surrounded the leper village, drove the lepers—men, women, and children—to the parade ground, where a pit had been prepared, with a thick layer of wood at the bottom. One by one the miserable wretches were forced to descend a ladder into this pit, and when all were seated on their funeral pile the soldiers poured a volley upon them from their rifles, a quantity of petroleum was thrown into the pit, and the lepers were exterminated by fire. The Kwangsi officials, we are informed, are in nowise ashamed of this dastardly deed. The following is from a translation of an official proclamation: "The lepers commit abominable excesses. . . . The recital of their crimes makes one's hair stand on end. I referred the matter to the President, who by a secret order commanded me to seize and kill all the lepers in Nanning. . . . Thus we are delivered for ever from their contagion. I assured myself of universal approval." This under the so-called Chinese Republic, in the Cantonese administrative area, within 500 miles of Hongkong!

### THE COTTAGE HOSPITAL REPORT.

A successful year is recorded in the latest annual report of the Purley Cottage Hospital, the supporters of which managed to organise a bazaar so successfully that over £450 was realised, with the gratifying result that a debt of £500 which had been hanging over the institution was extinguished by its means. Important staff changes have taken place, Sir Horace Brooks Marshall has accepted the office of president, and Mr. Douglas, who had to give up the secretaryship, has been succeeded by Mr. H. O. Monkley as honorary secretary to the institution. We are very glad to note an increase in church collections. The problem of making the cottage hospital report attractive is always a difficult one, since the question of expense enters in more necessarily than in the case of larger institutions. But we think that this report contains sufficient evidence of local interest, judging from the gifts there recorded, to justify the inclusion of at least one illustration. The general rule adopted by the best administered institutions is to include a photograph of the latest improvement, capable of being shown in an illustration, which has been carried out. Doubtless next year Mr. Monkley and his committee will turn their attention to this small but really important matter. An illustration is generally enough to gain at least one glance from any reader, who will not hesitate to throw mere letterpress into the waste-paper basket.

### THE DRUG BILL IN A LARGE HOSPITAL.

The pharmacist to Guy's Hospital, Mr. Finnemore, compiles annually a most interesting comparative summary of the principal drugs and dressings used in the service of the hospital; and the editor of the *Guy's Hospital Gazette* very wisely publishes it. Under the column "Antiseptics," for instance, we learn that lysol, hydrogen peroxide, and carbolic acid were rather more largely used in 1912 than in the previous year; whereas cresol, boric acid, formaldehyde, and iodoform were less extensively purchased. Mercuric antiseptics were practically stationary. As for anæsthetics, chloroform decreased slightly, and ether correspondingly increased; about six times as much of the latter is used as of the former. Absolute alcohol is rapidly approaching extinction, as far as its anæsthetic uses are concerned; and ethyl chloride has been in slightly less demand. Cocaine shows a small increase, as does eucaine also; novocain has a big jump however. But it is clear that local anæsthesia is not making any very exciting progress at Guy's. Among the sera purchased, we note that anthrax disappears from the list; and diphtheria antitoxin has been cut down to about two-thirds of the cost for 1911. Antistreptococcus serum also shows a heavy fall, as also do anti-colon serum, antitetanic serum, and normal horse serum. Probably vaccine therapy accounts for some of these falls. Pituitary extract has been more extensively employed; salvarsan and neo-salvarsan to the value of £70 have been used, and liquid paraffin to the value of £73. Tablets are up 100 per cent., and tinctures, syrup, and collodions are down by fairly substantial amounts.

### THIS WEEK'S DRUG MARKET.

Of chief interest is a decline in the quotations for cod-liver oil, notwithstanding that the results of the fishing in Norway are again reported to be poor; the reason for the decline is that buyers hesitated to make purchases at the figures recently ruling. The advance in the price of opium is well maintained. There is no alteration in the quotations for morphine and codeine, but the price of apomorphine has been reduced. It is not altogether improbable that the price of glycerine will be advanced. Eserine is lower owing to a reduction in the cost of the raw material, Calabar beans. Apiol is dearer. Oil of orange continues scarce and dear. The high price of essence of lemon is well maintained. Orris root is dearer. There is no change in the position of quinine. A further reduction in the price of cocaine is not improbable owing to an oversupply of raw materal. Cape aloes are somewhat higher in price. Cardamoms are rather lower, and sarsaparilla is dearer. Generally speaking, the market is unusually dull for the time of the year, notwithstanding the undoubted increase in the consumption of drugs occasioned by the operation of the Insurance Act.

### TO OUR READERS.

Contributions are specially invited from any of our readers to these columns. They should deal with topical subjects and news. They must be authenticated for the information of the Editor only. The minimum payment if published is 5s. There is no hard-and-fast rule as to space, but notes of about twenty lines in length are preferred.

